# Patients who achieved long-term clinical complete response and subsequently terminated multidisciplinary and anti-HER2 therapy for metastatic breast cancer: A case series

**DOI:** 10.1016/j.ijscr.2018.10.008

**Published:** 2018-10-12

**Authors:** Haruko Takuwa, Wakako Tsuji, Fumiaki Yotsumoto

**Affiliations:** Department of Breast Surgery, Shiga General Hospital, 5-4-30, Moriyama, Moriyama-city, Shiga, 524-8524, Japan

**Keywords:** HER2, human epidermal growth factor receptor 2, cCR, clinical complete response, T, trastuzumab emtansine, MBC, metastatic breast cancer, FISH, fluorescence in situ hybridization, CT, computed tomography, LHRH-a, luteinizing hormone-releasing hormone agonist, PTX, paclitaxel, Anti-HER2 therapy, Clinical complete response, Metastatic breast cancer, Multidisciplinary therapy, Termination of therapy

## Abstract

**Background:**

Human epidermal growth factor receptor 2 (HER2) -positive breast cancers tend to be more aggressive and more likely to recur than HER2-negative breast cancers. However, novel anti-HER2 therapies have dramatically improved the prognosis of patients with HER2-positive breast cancer.

**Case report:**

We review the cases of 4 women with metastatic breast cancer who achieved clinical complete response (cCR) and terminated their systemic therapy. Two patients had de novo metastatic disease and two patients experienced relapse after adjuvant therapy. All patients achieved cCR using multidisciplinary therapy, experienced prolonged complete remission, and subsequently terminated their systemic therapy without experiencing secondary recurrence.

**Conclusion:**

There is no evidence that systemic therapy can be safely terminated after a specific time period, although adverse events (e.g., cardiotoxicity) and unnecessary treatment should be avoided. Thus, it is possible that select patients may be suitable for termination of systemic therapy after they have achieved a prolonged period of cCR.

## Highlights

• Breast cancers that are positive for human epidermal growth factor receptor 2 (HER2) are aggressive and typically associated with a poor prognosis.
• Novel anti-HER2 therapies have recently improved the outcomes in these cases.
• We report a case series in which women were treated for metastatic HER2-positive breast cancer using trastuzumab and various chemotherapies.
• These patients ultimately achieved clinical complete response, and subsequently terminated their systemic therapy after maintenance therapy.
• Our findings indicate that select patients may be suitable for treatment termination if they have achieved a prolonged period of complete response.

## 1 Introduction

Overexpression of human epidermal growth factor receptor 2 (HER2) occurs in approximately 20–30% of breast cancers and leads to an aggressive disease with a poor prognosis [1,2]. However, novel anti-HER2 therapies, such as humanized monoclonal antibodies to HER2 (pertuzumab and trastzumab) and an antibody-drug conjugate (trastuzumab emtansine; T-DM1), have significantly improved progression-free survival and overall survival among patients with metastatic breast cancer (MBC) [[3], [4], [5], [6], [7]]. Furthermore, recent clinical trials included anti-HER2 therapy have indicated that 10–20% of patients with MBC can achieve a clinical complete response (cCR) [[4], [5], [6], [7], [8], [9]], although these treatments are associated with elevated risks of cardiac toxicities, such as congestive heart failure and left ventricular ejection fraction decline [[10], [11], [12], [13], [14], [15]]. This report examines 4 cases in which patients with MBC achieved long-term remission after anti-HER2 therapy and subsequently terminated their systemic therapy.

## 2 Methods and results

### 2.1 Patients

We retrospectively reviewed the medical records of 171 patients with MBC who underwent surgical treatment at our institute between 2011 and 2017. The retrospective protocol was approved by the appropriate ethics review board, and the study complied with the tenets of the Declaration of Helsinki.

The patients had either de novo MBC, local recurrence of breast cancer, or distant metastases that appeared after treatment of the primary cancer. Forty patients (23.4%) had a primary tumor that was positive for HER2, although 5 patients (2.9%) had primary HER2-negative disease and metastases that were HER2-positive (1 turned from HER2 score 0, and 4 turned from 2+, FISH negative). Table 1 shows the characteristics of the patients with HER2-positive metastatic or recurrent breast cancer. Cases with cCR were identified based on no evidence of disease after treatment for MBC (i.e., no clinical or radiological evidence of disease according to the Response Evaluation Criteria in Solid Tumors). These assessments were performed at frequencies and intervals that were selected by the treating physician, using computed tomography (CT), magnetic resonance imaging, and/or positron emission tomography. Nine patients achieved cCR, with 4 patients (Case 1–4) experienced distant metastasis and 5 patients experienced regional recurrence. Since five patients with regional recurrence obtained cCR by local resection, detailed information about them is omitted this report. The research work has been reported in line with the PROCESS criteria [16].

**Table 1:** Patient and disease characteristics of the HER2-positive metastatic or recurrent breast cancer patients.

Variable	all patients (n = 45)	Luminal-HER2 (n = 24)	HER2-enriched (n = 21)	p
Follow-up period (months) Median Range	41 0–135	44 7–135	38 0–110	0.622
Age at primary breast cancer (y.o.) Median Range	54 32–76	54 35–76	54 32–71	1.000
Age at metastatic breast cancer (y.o.) Median Range	56 32–77	57 36–77	56 32–72	0.826
Disease stage at diagnosis, No. (%) Stage 0 Stage I Stage II Stage III Stage IV Unknown	1 (2.2) 6 (13.3) 9 (20.0) 16 (35.6) 12 (26.7) 1 (2.2)	0 (0.0) 1 (4.1) 7 (29.2) 9 (37.5) 7 (29.2) 0 (0.0)	1 (4.8) 5 (23.8) 2 (9.5) 7 (33.3) 5 (23.8) 1 (4.8)	0.164
Histology, No. (%) Invasive ductal Other	42 (93.3) 3 (6.7)	23 (95.8) 1 (4.2)	19 (90.5) 2 (9.5)	0.472
HER2 status, No. (%) HER2 2+, FISH amp HER 3+	6 (3.3) 39 (86.7)	5 (20.8) 19 (79.2)	1 (4.8) 20 (95.2)	0.114
Ki-67 labeling index Median, SD Range	27.1 ± 20.5 2.0–90	20.2 ± 14.8 2.0–50	34.0 ± 23.5 5 - 90	0.087
Site No. of metastasis / recurrence 1 2 ≥ 3	15 (33.3) 13 (28.9) 17 (37.8)	9 (37.5) 6 (25.0) 9 (37.5)	6 (28.6) 7 (33.3) 8 (38.1)	0.764
No. of visceral metastasis / recurrence 0 1 2 3	51 (29.8) 76 (44.5) 40 (23.4) 4 (2.3)	8 (33.3) 10 (41.7) 4 (16.7) 2 (8.3)	6 (28.6) 9 (42.8) 5 (23.8) 1 (4.8)	0.900
Prior systemic therapy Anthracycline Other chemotherapy Trastuzumab Endocrine therapy	16 (35.6) 9 (20.0) 12 (26.7) 17 (37.8)	10 (41.7) 3 (12.5) 5 (20.8) 15 (62.5)	6 (28.6) 6 (28.6) 7 (33.3) 2 (9.5)	**0.018**

### 2.2 Case 1

**Fig. 1:**
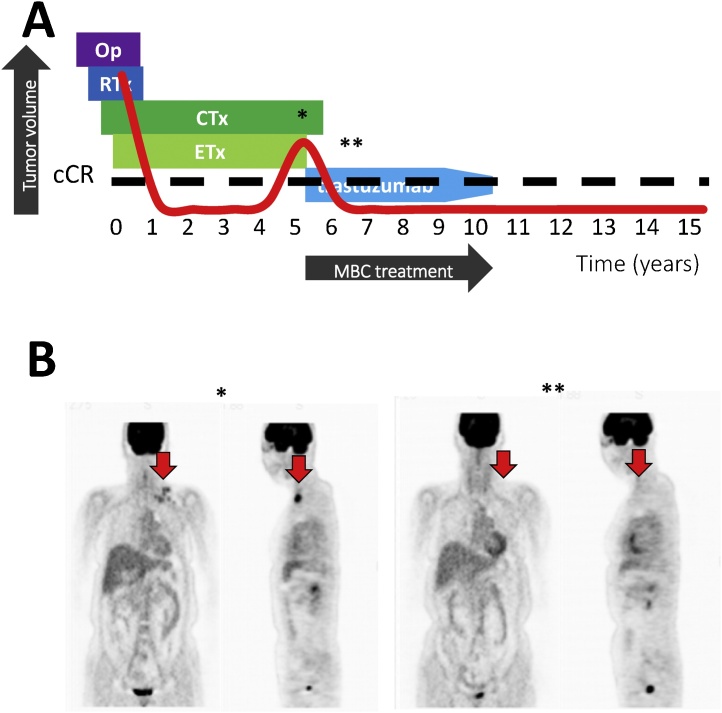
Image of the clinical course in case 1, a luminal-HER2 type breast cancer patient. (A) The history of breast cancer treatment. (B) The images show (*) when recurrence detected, and (**) radiologically complete remission. Ope = operation; RTx = radiotherapy; CTx = chemotherapy; ETx = endocrine therapy; cCR = clinical complete response; MBC = metastatic breast cancer.

A 41-year-old woman underwent breast conserving surgery and axillary dissection in February 2002. The Pathological results revealed that she had pT2N2M0 disease (stage IIIA, luminal-HER2 type breast cancer). The patient underwent postoperative chemotherapy using 4 cycles of 5-fulorouracil plus epirubicin plus cyclophosphamide. As trastuzumab had not been approved as an adjuvant therapy in Japan at that time, the patient also received luteinizing hormone-releasing hormone agonist (LHRH-a) with tamoxifen and tegafur plus uracil after the chemotherapy and whole-breast radiotherapy. At 4 years after surgery, and during adjuvant systemic therapy, she experienced recurrence in multiple supraclavicular lymph nodes. Thus, first-line treatment for MBC was started using paclitaxel (PTX; 80 mg/m^2^ on days 1, 8, and 15) and trastuzumab (4 mg/kg as a loading dose followed by 2 mg/kg as a weekly maintenance dose). After 4 cycles of the first-line treatment, the patient achieved a complete radiological response and a non-pathological values for CEA and CA15-3. The patient remained in cCR during 5 years of maintenance therapy using trastuzumab, and subsequently terminated systemic therapy. The last follow up was August 2018 and she has survived for 11.5 year after termination of anti-HER2 therapy (Fig. 1).

### 2.3 Case 2

A 41-year-old woman with cT3N2M0 disease (stage IIIA, luminal-HER2 type cancer) underwent preoperative chemotherapy using 2 cycles of epirubicin plus cyclophosphamide followed by 2 cycles of weekly PTX in 2013. Mastectomy and axillary lymph node dissection revealed a Grade 1b therapeutic effect. The association between pathological complete response and long-term outcomes was strongest in patients with triple-negative breast cancer and in those with HER2-positive, hormone-receptor-negative tumors who received trastuzumab [17]. However, the impact of pathological CR on luminal-HER2 type breast cancer patients is currently unknown.

**Fig. 2:**
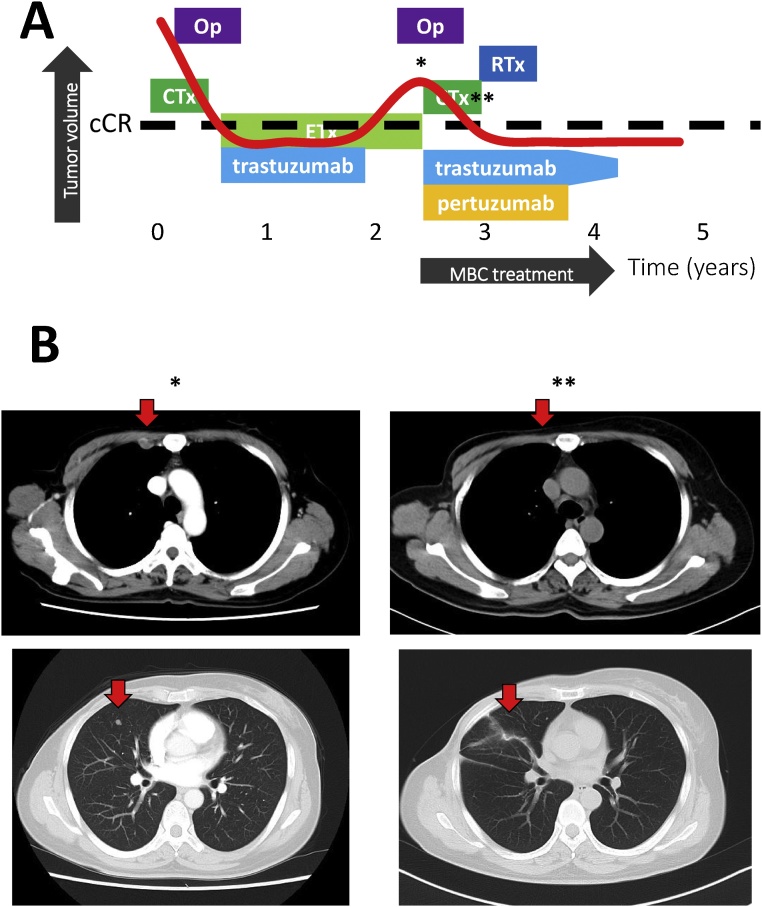
Image of the clinical course in case 2, a HER2-enriched type breast cancer patient. (A) The history of breast cancer treatment. (B) The images show (*) when recurrence detected, and (**) radiologically complete remission. Scar remained in her right lung because the patient received video assisted thoracic surgery as biopsy from metastatic site. Op = operation; RTx = radiotherapy; CTx = chemotherapy; cCR = clinical complete response; MBC = metastatic breast cancer

The patient subsequently received trastuzumab and LHRH-a with tamoxifen, but did not undergo post-mastectomy radiotherapy. At 2 years after surgery, and during adjuvant endocrine therapy, pathology results revealed lung and internal mammary lymph nodes metastases. Thus, first-line treatment for MBC was started using docetaxel (75 mg/m^2^ on day 1) with pertuzumab (840 mg as a loading dose followed by 420 mg on day 1 of each subsequent cycle) and trastuzumab (8 mg/kg followed by 6 mg/kg on day 1). After 4 cycles of the first-line therapy, the patient achieved a complete radiological response and a non-pathological values for CA15-3 and NCC-ST-439. She subsequently underwent irradiation to the chest wall and internal mammary lymph node region, and received maintenance therapy using pertuzumab plus trastuzumab for approximately 18 months. She stopped maintenance therapy at October, 2017. The last follow up was August, 2018 and she has survived for 10 months after termination of anti-HER2 therapy (Fig. 2).

### 2.4 Case 3

**Fig. 3:**
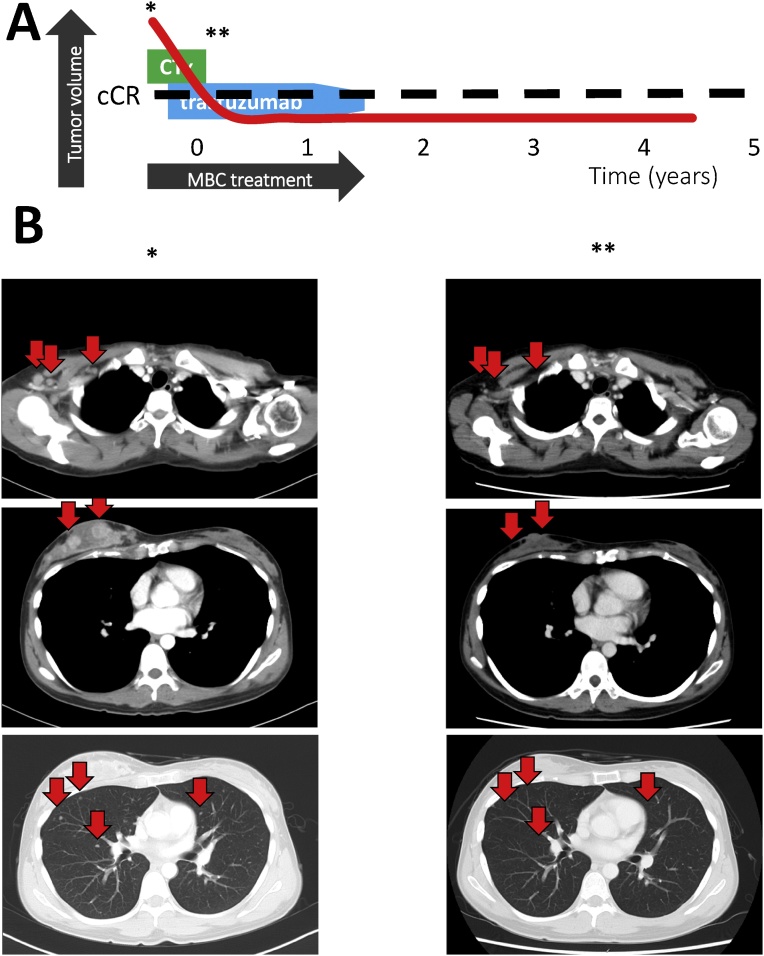
Image of the clinical course in case 3, a HER2-enriched type breast cancer patient. (A) The history of breast cancer treatment. (B) The images show (*) when the patient was diagnosed with MBC, and (**) radiologically complete remission. The patient did not receive surgical recession of tumor region. Op = operation; RTx = radiotherapy; CTx = chemotherapy; cCR = clinical complete response; MBC = metastatic breast cancer.

A 32-year-old woman was diagnosed with cT3N3M1 disease (HER2-enriched breast cancer), and multiple lung metastases were detected in CT in 2014. Docetaxel with pertuzumab and trastuzumab was not approved as a first-line treatment in Japan at that time. Radiological evaluations revealed no therapeutic effect from 2 cycles of first-line treatment using epirubicin (90 mg/m^2^) plus cyclophosphamide (600 mg/m^2^). Thus, weekly PTX and trastuzumab were administered as second-line therapy, and the patient achieved cCR after 4 cycles. She continued maintenance therapy using trastuzumab for 1 year and subsequently terminated her therapy at December, 2015. The last follow up was June 2018 and she has survived for 2 years and a half month after termination of anti-HER2 therapy (Fig. 3).

### 2.5 Case 4

**Fig. 4:**
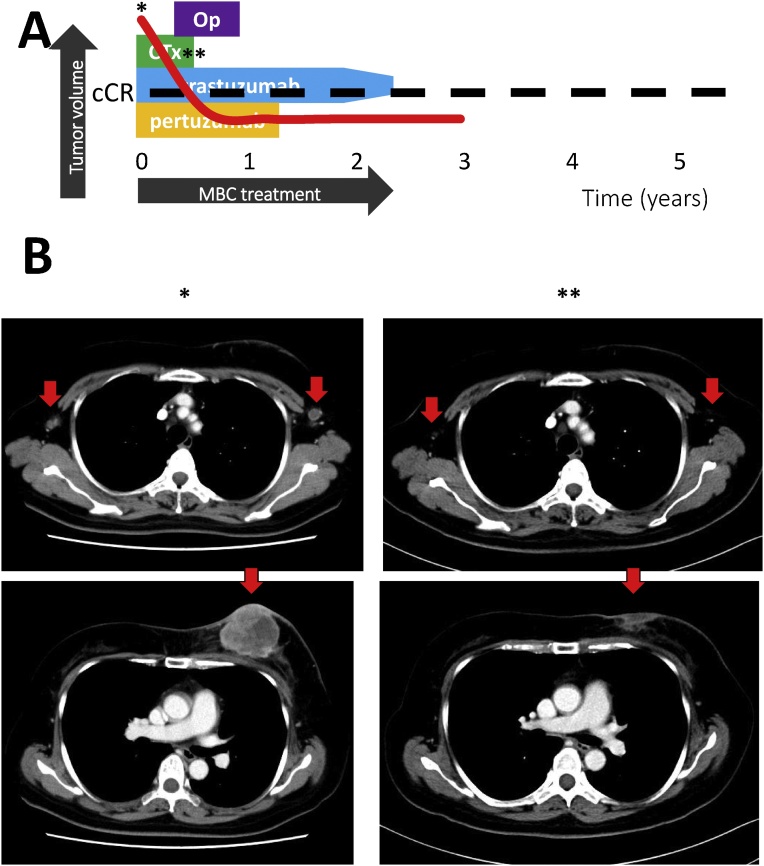
Image of the clinical course in case 4, a HER2-enriched type breast cancer patient. (A) The history of breast cancer treatment. (B) The images show (*) when the patient was diagnosed with MBC, and (**) radiologically complete remission. The patient received mastectomy and sentinel lymph node biopsy, proving pathological complete response in the left breast. (C) Tapering trastuzumab for termination after maintenance therapy. The dose of trastuzumab was gradually reduced in all patients. Op = operation; RTx = radiotherapy; CTx = chemotherapy; cCR = clinical complete response; MBC = metastatic breast cancer

A 56-year-old woman with cT4bN2M1 disease (HER2-enriched breast cancer) had contralateral lymph node metastasis that was pathologically detected in 2016. The patient started first-line treatment using docetaxel with pertuzumab and trastuzumab, achieved cCR after 4 cycles. Although the tumor disappeared from her left chest, an abscess-like secretion persisted from a skin ulcer. Mastectomy and sentinel lymph node biopsy were performed, and confirmed a pathological complete response. The patient continued maintenance therapy using pertuzumab and trastuzumab, but subsequently terminated systemic therapy after approximately 18 months at November, 2017. The last follow up was July, 2018 and she has survived for 8 months after termination of anti-HER2 therapy (Fig. 4).

## 3 Discussion

Despite the use of novel anti-HER2 therapies in recent decades, adverse events, such as cardiac toxicity, remain an important consideration during systemic therapy [[10], [11], [12], [13], [14], [15]].

**Fig. 5:**
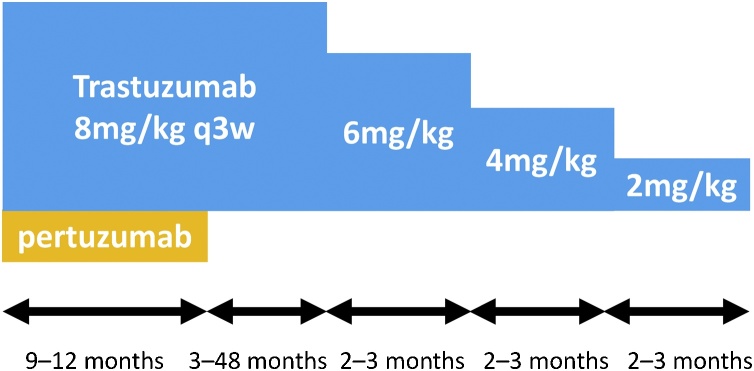
Tapering trastuzumab for termination after maintenance therapy.

The patients in Cases 1 and 2 experienced relapse during adjuvant therapy, and resistance to endocrine therapy was predicted. The patient in Case 1 had never received trastuzumab, while the patient in Case 2 had received trastuzumab without chemotherapy as postoperative therapy [4,5,[18], [19], [20]]. Both patients subsequently achieved cCR during their first-line therapy for MBC. The patients in Cases 3 and 4 were diagnosed with MBC, but subsequently achieved cCR using anti-HER2 therapy combined with chemotherapy (during second-line therapy in Case 3 and during first-line therapy in Case 4) [4,5,19]. All 4 patients subsequently terminated their systemic therapy for MBC. Interestingly, all patients had a relatively small total tumor volume, were asymptomatic during the systemic therapy, and only had 1–2 metastatic sites. After the cCR was confirmed clinically or pathologically, they terminated the chemotherapy and received maintenance anti-HER2 therapy for 1–4 years without any recurrent lesions being detected. How long the appropriate maintenance period is an important theme discussed among experts. Previous reports about HER2-positive metastatic breast cancer patients who achieved cCR was referred [[20], [21], [22], [23], [24], [25], [26], [27]]. Furthermore, all patients underwent tapering of maintenance therapy dose (Fig. 5), as even low-dose trastuzumab is known to have an antitumor effect [[28], [29], [30], [31]]. If the tumor recurs or regrows during this low-dose trastuzumab period, the cancer cells are resistant to anti-HER2 therapy. However, if patients terminate treatment and experience a long recurrence-free period, it is possible that the cancer may remain susceptible to re-challenge using anti-HER2 therapy [18].

In the previous studies, median OS of HER2-positive breast cancer patients was under 40 months even after the new target agents [4,[6], [7], [8], [9],^11^,^32^,^33^], Pertuzumab and T-DM1 treatment. Patients surviving over 4 years are thought to relatively longer prognosis. For these patients, it is urgent to avoid complications that contribute to the treatment as well as prolonging their prognoses. In general, 12 months of adjuvant trastuzumab therapy is the standard treatment duration for patients with HER2-enriched disease, even with locally advanced breast cancer when they achieved pathological CR after 6 months of preoperative chemotherapy. These patients undergo treatment-free follow-up after a year maintenance trastuzumab. Patients with de novo stage IV breast cancer or postoperative recurrent disease may have more tumor volume than those with early breast cancer patients and distant metastasis associates with complicated mechanisms. Thus, we believe that the maintenance duration in MBC should be longer than postoperative adjuvant therapy.

The initial treatment is considered the most important for patients with MBC. However, if the case does not involve de novo stage IV disease, it is possible that the patient may have been treated heavily using anti-HER2 therapy and chemotherapy [[32], [33], [34], [35]]. Therefore, cCR is considered to be relatively difficult to achieve in cases of MBC, compared to de novo stage IV cancer. Although some reports have described patients achieving cCR, after treatment for metastatic HER2-positive breast cancer, we are not aware of any reports regarding the termination of systemic therapy for these patients. Thus, although intensive monitoring is needed after terminating therapy, it is possible that select patient may not need to continue receiving maintenance treatment for MBC.

## 4 Conclusions

The present cases highlight the possibility that select patients with MBC may be able to terminate systemic therapy after they have achieved a prolonged period of cCR.

## Conflicts of interest

The authors declare that they have no competing interests.

## Funding

This research did not receive any specific grant from funding agencies in the public, commercial, or not-for-profit sectors.

## Ethics approval

Our institution has exempted ethical approval for this case series as there are no patient identifiers in the images or text.

## Consent

Written informed consent was obtained from the patients for publication of this case series and any accompanying images.

## Authors' contributions

HT, WT, FY participated in the treatment, data interpretation and manuscript preparation. HT wrote and edited the manuscript. All authors read and approved the final manuscript.

## Registration of research studies

researchregistry4166.

## Guarantor

Haruko Takuwa

## Provenance and peer review

Not commissioned, externally peer reviewed.
